# Growth Performance, Nutrient Digestibility, Blood Profiles, Excreta Microbial Counts, Meat Quality and Organ Weight on Broilers Fed with De-Oiled Lecithin Emulsifier

**DOI:** 10.3390/ani10030478

**Published:** 2020-03-12

**Authors:** Xiao Liu, Seo Bin Yoon, In Ho Kim

**Affiliations:** Department of Animal Resource and Science, Dankook University, Anseodong, Cheonan, Choongnam 330-714, Korea; xiaoliu249@foxmail.com (X.L.); syoon@feedbest.co.kr (S.B.Y.)

**Keywords:** emulsifier, de-oiled lecithin, growth performance, blood profile, broiler

## Abstract

**Simple Summary:**

Commercial broilers have a short production cycle and a high requirement for energy, so the need to add lipids to the diet of broilers is inevitable. Adding an exogenous emulsifier to diets can enhance the digestion and absorption of lipids in broiler chickens. The effect of de-oiled lecithin as emulsifier on performance, lipid blood profiles, nutrient digestibility, organ weight, meat quality, and excreta microbial counts in broilers were evaluated in the present study. It was determined that the de-oiled lecithin supplemented diets improved the digestion and absorption of lipids through its emulsification, thus promoting better growth performance and health conditions of broilers. The results of this research provide a theoretical basis and a new insight for the applications of lecithin as a feed additive in the poultry feed industry.

**Abstract:**

This research evaluated the effects of de-oiled lecithin (DOL) as an exogenous emulsifier in broilers. Totally, 480 male broilers (1-d-old, Ross308) were raised for a 35-day feeding experiment. Broilers were randomly divided into three dietary groups including the addition of 0, 61.80%de-oiled lecithin (DOL-60), 97.16%de-oiled lecithin (DOL-97) into the basal diet. Broiler chickens fed with DOL-60 and DOL-97diets had greater body weight gain (BWG) during 1–7 days, 8–21 days, and the overall experimental period (*p*< 0.05),greater(*p* < 0.05) breast muscle percentages, and lower (*p* < 0.05) low-density lipoprotein cholesterol (LDL/C) concentrations. Furthermore, broiler chickens fed with DOL-97 diet showed the highest (*p*< 0.05)BWG during 22–35 days and feed intake during 8–21 days, lowest (*p*< 0.05) feed conversion ratio during 22–35 days and overall period, highest (*p*< 0.05) concentration of serum high-density lipoprotein-cholesterol (HDL/C), lowest (*p* < 0.05) concentration of serum low-density lipoprotein-cholesterol (LDL/C),excreta population of *Escherichia coli (E. coli)*, and highest(*p* < 0.05) value of breast muscle redness. In summary, broiler diets inclusion of DOL-97 decreased the excreta *E. coli* counts, improved the growth performance, increased breast muscle percentage and redness, and enhanced concentrations of serum HDL/C and LDL/C.

## 1. Introduction

Commercial broilers have a short production cycle and a high requirement for energy. Therefore, the need to add lipids to their diet is inevitable. Lipids (fats and oils) contain the highest level of calories among all nutrients, and mainly provide the energy for animals. It is well known that dietary supplementation of animals with fats and vegetable oils can improve growth performance and help to achieve industry standards in poultry [1]. Currently, the use of different sources of fat, such as soybean oil, black soldier fly larvae fat, canola oil, and tallow, is common in broiler nutrition [2,3,4]. Tallow is considered to be one of the most important raw materials [5]. The energy metabolism of newly hatched chickens changed dramatically, from yolk-based lipid supply to exogenous carbohydrate-based feed source [6]. However, the bile salt and lipase production and secretion are limited especially in young animals, which results in a weak digestion and absorption of lipids [7,8]. Adding emulsifiers to the diets can be used as an approach to overcome this limitation.

Lecithin is a natural fatty substance found in many foods, including soybean, egg yolk and whole grain. Soy-lecithin is a by-product of soybean oil processing, and it mainly contains phosphatides such as 19–21% phosphatidylcholine, 20–21%inositol phosphatides, 8–20%phosphatidyl-ethanolamine and 5–11% other phosphatides [9]. Besides being a source of energy, it also serves as an emulsifier by modifying or binding phospholipid molecules [10,11,12]. Emulsifiers include hydrophilic and hydrophobic molecules, which can distribute oil droplets evenly in the emulsion. In such cases, emulsifiers can enhance the absorption and digestion of lipids [13]. Positive effects on utilization and digestibility of nutrients in animals were observed by previous studies with the addition of lecithin as emulsifier [14,15,16]. Furthermore, Wang et al. indicated that the digestibility of dry matter, protein and fat was improved with supplementation of lecithin in broiler diets [17]. In the production of high-purity lecithin products, crude lecithin needs to be de-oiled [18]. De-oiled lecithin (DOL) is a powdery lecithin derived from crude soybean lecithin by industrial methods, including filtration, bleaching (hydrogen peroxide) and solvent extraction (acetone). Its main components are similar to crude soybean lecithin, but the content is obviously higher than crude soybean lecithin, thereby it may show better emulsifying ability. However, few studies have evaluated the use of DOL in broilers.

It was hypothesized that DOL as an exogenous emulsifier could have a beneficial effect in emulsification, thereby enhancing the absorption and performance of broilers fed on tallow diet. Therefore, the principal objective of the current research was to investigate the effects of DOL as an emulsifier on growth performance, apparent total tract digestibility, blood profiles, meat quality, organ weight, and excreta microbial counts in broilers.

## 2. Materials and Methods

The management and care of animal experimental protocols described in this research were assessed and accepted by the Dankook University Use Committee and Animal Care (Case No. DK-1-1717), Republic of Korea.

### 2.1. Source of De-Oiled Lecithin

The commercial DOL product used in this experiment was obtained from a local company (Feed BEST Co., Ltd., Cheonan, Korea). The commercial DOL powder was produced through filtration, bleaching (hydrogen peroxide) and solvent extraction (acetone) process from crude liquid lecithin, which was degummed and refined from soybean oil. The purity of the de-oiled lecithin-60 (DOL-60) and de-oiled lecithin-97 (DOL-97) were 61.80% and 97.16%, respectively. Table 1 shows the phospholipid compositions of DOL-60 and DOL-97.

### 2.2. Experimental Design, Animals, Diets and Housing

Totally, 480 (1-d old Ross 308, male) broilers with an average initial body weight of 41.95 ± 1.00 g were used in 35 days feeding experiment. The experiment was conducted in 3 phases consisting of phase 1 (1–7 days), phase 2 (8–21 days) and phase 3 (22–35 day). Broilers were randomly allotted to 3 dietary treatments containing the addition of 0, 61.80%de-oiled lecithin (DOL-60), 97.16%de-oiled lecithin (DOL-97) into the basal diet. All the diets were presented in mash form, and formulated to meet or exceed the NRC (1994) [19] requirement for broilers (Table 2). 

The DOL-60 and DOL-97were mixed properly using a mixer (DDK-801F, Daedong Tech, Anyang, Korea). Due to the low inclusion of DOL (0.1%), it was not indicated the energy content of DOL in the formulation. There were 10 replicates per treatment with 16 broilers per replicate. The temperature of the broilers rooms were controlled at 33 ± 1 °C for the first 3 days, and then decreased by 3 °C per week bit by bit to 24 °C until the end of the trial and humidity was maintained around 60% throughout the entire experiment. Artificial lighting was provided by fluorescent lamps 24 h a day. Each cage has two feeders and two nipple drinkers in each side, and the feed and water were freely accessed during the experiment.

### 2.3. Sampling and Measurements

The birds and feeders were weighed on d 0, 7, 21, and 35 to allow calculations of feed conversion ratio (FCR), feed intake (FI), and body weight gain (BWG). From d 28–35, apparent total tract digestibility (ATTD) of nitrogen (N), energy, and dry matter (DM) was evaluated by adding 0.2 %chromic oxide to the diets as an indigestion marker. Fresh excreta samples were gathered from each cage during the last three days of experiment. All the fresh samples were stored at −20 °C for further analysis. Before the chemical analysis, the feed and excreta samples were defrosted and dried at 70°C for 72h in a forced-air oven (model FC-610, Advantec, Toyo Seisakusho Co. Ltd., Tokyo, Japan). After drying, the sample was ground to less than 1 mm screen size. Then, all samples were analyzed for N (method 968.06) [20], Ca (method 984.01) [21], P (method 965.17) [20], and fat (method 954.02) [21]. Crude protein was calculated as N × 6.25. Nitrogen content was measured by a machine (Kjeltec 2300 Nitrogen Analyzer; Foss Tecator AB, Hoeganaes, Sweden).Energy was determined by Parr 6400 oxygen bomb calorimeter (Parr Instrument Co., Moline, IL, USA). Chromium was determined by a machine (Shimadzu UV-1201, Shimadzu, Kyoto, Japan). The ATTD of nutrients was then calculated using the formula: digestibility (%) = {1 − [(Nf × Cd)/ (Nd × Cf)]} × 100, where Cf = chromium concentration in feces (% DM), Cd = chromium concentration in diet (% DM), Nf = nutrient concentration in feces (% DM), and Nd = nutrient concentration in diet (% DM).

On day 35, two chickens per cage (total of 10 per treatment) were randomly chosen and bled for collecting blood samples from the wing vein into vacuum tubes (Becton Dickinson Vacutainer Systems, Franklin Lakes, NJ, USA).Blood samples were centrifuged at 3000× *g* for 20 min at 4°C. After that the serum samples were stored at –20°C until further analysis. The serum concentration of triglyceride, high-density lipoprotein cholesterol (HDL/C), total cholesterol, and low-density lipoprotein cholesterol (LDL/C) were analyzed by a blood analyzer (Advia 120, Bayer, Tarrytown, NY, USA).

The chickens for blood collection were weighed and slaughtered. A trained person removed the breast meat, gizzard, bursa of Fabricius, spleen, liver, and abdominal fat. All samples were patted dried to remove excess moisture and were weighed for determining the relative organ weight percentage. A machine (Konica Minolta Sensing Inc., Osaka, Japan) was used for hunter lightness (L*), yellowness (b*), and redness (a*), of breast meat. The water holding capacity (WHC) was determined following the method described by Kauffman et al. (1986) [22].The method of the percentage of drip loss was analyzed on day 1, 3, 5, and 7, which was described by Honikel (1998) [23]. Four gram meat sample was heated in a water bath at 70 °C for 30 min. After that, the cooking loss was calculated as the weight of the original sample minus the final cooked sample.

Two chickens per cage (10 birds each treatment) were chosen randomly, and excreta samples were collected from the cloacae and placed into micro-tubes by massaging the abdomen. One gram of the excreta sample was diluted with 9 mL of 1% peptone broth (Becton, Dickinson and Co., Franklin Lakes, NJ, USA) and homogenized. *Escherichia coli* (*E. coli*) and *Lactobacillus* were isolated by inoculation of MacConkey agar plates (Difco Laboratories, Detroit, MI, USA) and *Lactobacillus* medium agar plates (Medium 638, DSMZ, Braunschweig, Germany) with 10 times continuous diluent (1% peptone solution).The *Lactobacilli* medium agar plates and the MacConkey agar plates were then incubated for 48 h at 39 °C and 24 h at 37 °C under anaerobic conditions, respectively. The *E. coli* and *Lactobacillus* bacteria were counted immediately after being removed from the incubator.

### 2.4. Statistical Analyses

Data were subjected to analysis as a completely randomized design by using the GLM procedure of SAS (SAS Institute Inc., Cary, NC, USA). Each cage was considered as the experimental unit. Differences between treatments were detected by Tukey’s multiple range tests. The data were presented as the pooled standard error of means. The *p* < 0.05 was considered as significant.

## 3. Results

### 3.1. Growth Performance and Nutrition Digestibility

Effects of DOL-60 and DOL-97 on broilers’ performance and nutrient digestibility were shown in Table 3 and Table 4. 

During 1–7 days, DOL-60 and DOL-97 diets had no effects on FI and FCR (*p*> 0.05), but broilers in TRT1 and TRT2 presented an increased in BWG compared those fed with CON diet (*p* < 0.05).During 8–21 days, supplementation of DOL-60 and DOL-97 increased the birds’ BWG (*p* < 0.05), but had no influences in FCR compared with the broilers in CON group (*p* > 0.05). Additionally, diets supplementation with DOL-97 had higher BWG and FI than CON (*p* < 0.05). During 22–35 days, the birds fed with DOL-97 diet showed significant (*p* < 0.05) improvements in BWG and FCR than these birds in the CON treatment. During overall period, FI was not affected inDOL-60 and DOL-97treatments (*p* > 0.05). However, broilers in TRT1 and TRT2 had higher BWG (*p* < 0.05) than the CON diet; furthermore, birds fed DOL-97 diet had the lowest FCR (*p* < 0.05) among the three groups. However, no significant differences were observed in the ATTD of DM, N, energy, and fat among different dietary treatments (*p* > 0.05).

### 3.2. Blood Profiles

As shown in Table 5, dietary supplementation of DOL had no effects on serum total triglyceride and cholesterol in broilers (*p*> 0.05). The broilers fed with DOL-97 diet had a greater (*p*< 0.05) serum concentration of HDL/C than those fed CON diet. In addition, broilers in TRT1 and TRT2 treatments showed lower serum LDL-C concentrations compared with the birds in the CON group.

### 3.3. Meat Quality and Organ Weight

No differences were observed in pH, lightness value, and yellowness values, WHC, cooking loss, and drip loss in this study (*p*> 0.05). However, broilers fed with the DOL-97 supplementation diet had higher (*p*< 0.05) redness value than those fed the CON diet. 

Additionally, no differences were observed in the relative weight parameters such as liver, spleen, abdominal fat, bursa of Fabricius, and gizzard among the three dietary treatments (*p* > 0.05);whereas, broiler fed DOL-60and DOL-97 inclusion diets had the greater (*p* < 0.05)breast muscle percentage than the CON group(Table 6).

### 3.4. Excreta Microbial Counts

As shown in Table 7, no effects were observed in the excreta population of *Lactobacillus* among the different dietary groups (*p* > 0.05). However, broilers in TRT2 group had a lower (*p* < 0.05) excreta *E. coil* counts compared with those birds fed with the CON diet.

## 4. Discussion

### 4.1. Growth Performance and Nutrition Digestibility

In the present study, the supplementation of DOL showed a positive influence on ADG and FCR in broilers fed with the diets containing tallow as the fat source. Because of the limited data on the using of DOL supplementation in broilers, we had to compare our finding with the results of the studies that used lecithin. Consistent with our research, Yang et al. reported that supplementation of lecithin positively affected the growth performance of broilers fed with the soybean oil diet [24]. Siyal et al. reported that ADG of broilers fed with the palm oil diet containing soy lecithin was increased during days 0–21, days 21–42, and days 0–42 and FCR decreased during days 0–42 [25]. Similarly, when lecithin was added to tallow diets of broilers, a substantial increase of body weight was obtained [14]. Mahmoudi et al. showed that FCR was improved with supplementation of soy lecithin in the diet of broilers [26]. Moreover, Jin et al. suggested that weanling pigs dietary containing lecithin and tallow improved growth performance [27]. The increased digestibility of fatty acid may possibly causes the improvement of growth performance, accelerate the emulsification of lipid in small intestine, and promote the activation of lipase [28]. In addition, exogenous emulsifier could promote the secretion of endogenous bile acid, and further improve the utilization rate of tallow. However, Blanch et al. showed that no effects of body weight was observed when roosters received food containing soybean lecithin and tallow [29]. In this study, the content of phosphatidylcholine of DOL-60 and DOL-97 is 31.71% and 38.24% respectively, which is much higher than that of crude soybean lecithin (19%–21%) [9]. DOL can give full play to its emulsifying effect, which is more conducive to the combination of nutrients and digestive enzymes, promote the absorption of nutrients, thus achieving better growth performance [30]. Therefore, the inconsistency results of these researches may be due to the differences in the types of lecithin, and gender of the chickens.

No significant influences were observed on the nutrient digestibility parameters; whereas, there have numerical increasing tendency on the fat digestibility in the broilers fed with DOL supplemented diets. The function mechanism of DOL could not be clearly established. Our earlier studies reported that dietary lecithin supplementation did not affect the DM digestibility in weaning piglets [31]. In broilers, Huang et al. indicated that the addition of lecithin to a diet that contained soy oil did not affect N digestibility [32]. Furthermore, improvement of fat digestibility via dietary inclusion of lecithin in chick fed the tallow diet has been documented by Polin [33] and Wang et al. [34]. Reis de Souza et al. indicated that the addition of lecithin and tallow in pig diet improved fat digestibility, but did not cause a difference in N or energy digestibility [35]. Jones et al. showed the fat (tallow) digestibility of weaning pigs increased with the addition of lecithin [36]. Soares and Lopez-Bote indicated that no differences were observed on fat digestibility when lecithin was added in diets containing lard or soybean oil in weaned piglets [31]. Young poultry exhibit lower fat digestion capacity due to the lower rate in synthesis and recirculation of bile salts [37]. At this time, the addition of DOL as an emulsifier to broilers diet could accelerate the combination of fatty acids and micelles, thus promoting the digestion of lipids. However, due to the short digestive tract and unstable digestibility of the broilers, the effect of increasing the fat digestibility in this study seems not obvious. In addition, these different results may be associated with the type of the fat source, or the type of the animal in the trial used.

### 4.2. Blood Profiles

Supplementing the diets with saturated fatty acids, such as lard or tallow, may resulting an increasing concentrations of blood serum lipids (such as LDL, HDL, cholesterol, and triglyceride) [38]. In theory, the supplementation of emulsifier could effectively use energy, and reduce the cholesterol, LDL and triglyceride concentrations and increase the HDL levels [38,39,40]. In the current study, it showed that DOL-97 increased the concentrations of HDL/C, and both DOL-60 and DOL-97 decreased the concentrations of LDL/C. In line with this study, Jones et al. demonstrated that LDL/C decreased when lecithin was fed as an emulsifier to weanling pigs [36]. Huang et al. reported that LDL/C was reduced by soy-lecithin, whereas HDL/C was improved in broiler chickens [40]. Similarly, Siyal et al. showed LDL/C concentrations were decreased in 0.1% soybean lecithin group in comparison with basal diet in broiler chickens [25]. Possibly, the increase of HDL/C concentrations may be due to the better emulsification of supplementation of DOL, which can effectively and fully use of fat. Blood serum HDL/C and LDL/C contents support the lipid metabolism information. HDL is involved in the reverse transport of cholesterol, helping to extract excess cholesterol deposited in the blood vessels walls and send it back to the liver, where it is expelled through the gastrointestinal tract [41]. Also, HDL has important anti-inflammatory properties [42], and it may inhibit harmful bacteria in the intestinal tract, such as *E. coli*, thereby promoting intestinal health. Meanwhile, HDL has an important antioxidant effect, which can not only inhibit the oxidation of phospholipids, but also reduce the activity of LDL [42]. A lower value of LDL/C is better because LDL can be oxidized to ox-LDL, and when LDL is in excess, the cholesterol it carries accumulates in the walls of the arteries [43].Lipid oxidation could lead to the inflammation [42]. In addition, the lower LDL/C and higher HDL/C help to keep blood vessels dilated, which promotes better blood flow [43]. These also indicated that DOL has a positive effect in promoting the health of broilers. However, Huang et al. indicated that triglyceride levels were significantly decreased when lecithin was added to the broilers diets [32]. In our study, DOL diet did not affect the concentrations of total cholesterol, and triglycerides. It is hypothesized that the inconsistent findings could be due to the age of broilers, the types of fat sources, and the types and levels of DOL in the diet. The exact mechanism by which DOL affects serum profile remains to be further studied.

### 4.3. Meat Quality and Organ Weight

Meat color is recognized as a parameter that could determine the acceptance of the product by consumers, which is due to the concentration of hemoglobin, cytochrome C, and myoglobin, their chemical states, and the light-scattering properties of the meat. In this study, the redness value of breast meat was increased by DOL-97 supplementation. However, Upadhaya et al. observed similar results that the redness value of meat also indicated a trend in improvement with the increase of emulsifier dosage [38]. The existence of myoglobin on the surface of meat may lead to the positive effects of redness value. It is due to the meat surface developing when myoglobin is oxidized, and a bright red color of freshly cut meat is given [44]. In terms of organ weight, DOL supplementation had no significant effect on the relative weight of spleen, bursa of Fabricius, liver, gizzard, and abdominal fat in the current study. Consistent with our research, Huang et al. showed that soy-lecithin did not significantly affect the percentage of abdominal fat and liver fat. However, the relative weight of the breast muscle increased in DOL diets [40]. Similarly, Huang et al. showed that dietary soy-lecithin supplementation had a higher percentage of thigh muscle fat compared with basic diets of broilers [40]. Zhang reported that an emulsifier added to broilers diets increased the percentage of fat in the breast and thigh [45]. It is consistent with our results and indicated that DOL as an exogenous emulsifier can improve the circulation of lipids in the broilers body, thereby improving the muscle quality.

### 4.4. Excreta Microbial Counts

The gastrointestinal system is the body’s largest organ for immune function [46]. In addition, overabundance of *E. coli* in the gastrointestinal system causes a diarrhea, and a decrease in the performance in animals [47]. Because *Lactobacillus* can produce a broad spectrum of bacterins and eliminate a variety of intestinal pathogens, they can improve the gut microbiota [48]. Therefore, *E. coli* and *Lactobacillus* are important indicators to reflect the gastrointestinal health. Excreta *E. coli* counts were lower for birds receiving DOL-97 diet compared with the basal diet, but no differences in excreta *Lactobacillus* counts were observed among dietary groups. However, to the best of our knowledge, there were currently limited researches about DOL in poultry to be directly compared. Based on our findings, the decreased harmful bacteria in the intestinal tract indicated the improvement of intestinal health. This may improve the vitality of the gastrointestinal system, enhance disease resistance, and resist the infection of pathogenic microorganisms of broilers. Thus indirectly promote the absorption of nutrients, then improving the growth performance. In addition, due to the anti-inflammatory properties of HDL, it may inhibit harmful bacteria in the intestinal tract, such as *E. coli*. Therefore, the improvement of HDL/C may also one of the reasons to explain the decrease of DOL supplementation on *E. coli* counts in the current study. Furthermore, the mode of actions of DOL on the fecal microflora of broilers remains to be further studied.

## 5. Conclusions

In conclusion, dietary supplementation with DOL-97 enhanced growth performance, increased blood serum HDL/C concentration, decreased serum LDL/C concentration and *E. coli* counts. Moreover, dietary DOL-60 and DOL-97 supplementation enhanced breast muscle in broilers. Therefore, DOL-97 has potential positive effects as a feed additive in broilers industry. More researches are needed to determine the mechanisms underlying the effect of DOL.

## Figures and Tables

**Table 1:** Phospholipid composition of DOL-60 and DOL-97.

Items,%	DOL-60	DOL-97
Phosphatidyl choline	31.71	38.24
1-lysophosphatidyl choline	0.25	0.53
2-lysophosphatidyl choline	2.53	5.33
Phosphatidylinositols	21.04	21.75
Phosphatidylserines-Na	1.02	0.90
Phosphatidylethanolamines	22.49	18.46
Lysophosphatidylethanolamines	1.44	2.15
Acylphosphatidylethanolamines	4.77	1.22
Phosphatidyl glycerol	1.97	1.83
Diphosphatidylglycerols	1.74	1.12
Phosphatidic acid	9.67	5.39
Lysophosphatidic acid	0.53	0.57
Other phosphatidylinositols	0.83	2.50

**Table 2:** Ingredient composition of experimental diets (basal diets)*.

Ingredients, %	Phase 1 (d 1–7)	Phase 2 (d 8–21)	Phase 3 (d 22–35)
Corn	49.67	56.31	63.74
Soybean meal	34.57	25.21	16.95
Corn gluten	0.87	-	-
Sesame meal	-	2.00	2.00
DDGS, corn	3.00	5.00	5.00
Meat meal	2.00	3.00	5.00
Tallow	5.60	4.50	3.80
Limestone	1.07	0.87	0.7
Mono-dicalcium phosphate	1.64	1.27	1.05
Salt	0.33	0.24	0.19
NaHCO_3_	-	-	0.02
Methionine (99%)	0.38	0.39	0.36
Lysine (50%)	0.54	0.73	0.73
Threonine (98.5%)	-	0.18	0.16
Viramin premix^†^	0.10	0.10	0.10
Choline (50%)	0.13	0.10	0.10
Mineral premix^‡^	0.10	0.10	0.10
Analyzed composition			
Dry matter	88.50	88.60	88.70
Moisture	11.50	11.40	11.30
Crude protein	22.50	20.50	18.50
Crude fat	7.90	7.40	7.20
Fiber	2.70	2.70	2.60
Ash	6.70	6.00	5.60
Metabolizable energy	3100	3149	3248
Calcium	1.00	0.90	0.90
Total Phosphorous	0.70	0.70	0.70
Lysine	1.50	1.40	1.20
Methionine	0.70	0.70	0.70
Cysteine	0.30	0.30	0.30
Threonine	0.80	0.90	0.80
Tryptophan	0.20	0.20	0.20

* The other dietary treatments were obtained by including 0.1% DOL in replacement of corn. ^†^ Provided per kg of complete diet: 11,025 IU vitamin A; 1,103 IU vitamin D3; 44 IU vitamin E; 4.4 mg vitamin K; 8.3 mg riboflavin; 50 mg niacin; 4 mg thiamine; 29 mg D-pantothenic acid; 166 mg choline; 33 μg vitamin B12.^‡^ Provided per kg of complete diet: 12 mg Cu (as CuSO_4_·5H_2_O); 85 mg Zn (as ZnSO_4_); 8 mg Mn (as MnO_2_); 0.28 mg I (as KI); 0.15 mg Se (as Na_2_SeO_3_·5H_2_O).

**Table 3:** The effect of dietary DOL supplementation on growth performance in broilers^†,‡^

Items	CON	TRT1	TRT2	SEM^4^	*p* Value
d 1 to 7					
BWG^3^, g	123^b^	132^a^	128^a^	1.83	0.0051
FI^3^, g	163	170	165	3.21	0.2588
FCR^3^	1.327	1.284	1.290	0.02	0.0901
d 8 to 21					
BWG, g	574^b^	595^a^	612^a^	7.13	0.0054
FI, g	870^b^	896^ab^	918^a^	12.77	0.0498
FCR	1.517	1.505	1.502	0.01	0.7098
d 22 to 35					
BWG, g	996^b^	1016^ab^	1022^a^	8.45	0.0499
FI, g	1690	1686	1674	11.57	0.6695
FCR	1.698^a^	1.660^ab^	1.639^b^	0.02	0.0452
Overall					
BWG, g	1692^b^	1744^a^	1761^a^	8.28	<0.0001
FI, g	2723	2752	2758	20.39	0.4539
FCR	1.609^a^	1.578^ab^	1.566^b^	0.01	0.0428

^†^ Abbreviations: CON, basal diet; TRT1, basal diet + 0.1% DOL–60, and TRT2, basal diet + 0.1% DOL–97; BWG, body weight gain; FI, feed intake; FCR, feed conversion ratio. ^‡^ Standard error of the mean. ^a,b^ Means in the same row with different superscripts differ by the Tukey test (*p* < 0.05).

**Table 4:** The effect of dietary DOL supplementation on apparent total tract digestibility in broilers ^†,‡.^

Items, %	CON	TRT1	TRT2	SEM^3^	*p* Value
Dry matter	72.77	74.06	74.29	0.90	0.4580
Nitrogen	68.50	70.27	70.59	0.96	0.2857
Energy	73.45	75.52	75.98	0.86	0.1229
Fat	77.80	80.07	81.38	0.98	0.0610

^†^ Abbreviations: CON, basal diet; TRT1, basal diet + 0.1% DOL–60, and TRT2, basal diet + 0.1% DOL–97. ^‡^ Standard error of the mean.

**Table 5:** The effect of dietary DOL supplementation on blood profiles in broilers ^†,‡^.

Items	CON	TRT1	TRT2	SEM^3^	*p* Value
Cholesterol, mg	140	149	145	2.84	0.1133
Triglyceride, mg	83	87	91	2.78	0.1842
High-density lipoprotein cholesterol, mg/dl	101^b^	106^ab^	109^a^	1.87	0.0267
Low-density lipoprotein cholesterol, mg/dl	43^a^	33^b^	33^b^	2.87	0.0409

^†^ Abbreviations: CON, basal diet; TRT1, basal diet + 0.1% DOL-60, and TRT2, basal diet + 0.1% DOL-97. ^‡^ Standard error of the mean. ^a,b^ Means in the same row with different superscripts differ by the Tukey test (*p* < 0.05).

**Table 6:** The effect of dietary DOL supplementation on meat quality in broilers ^†,‡^.

Items	CON	TRT1	TRT2	SEM^4^	*p* Value
pH value	5.48	5.44	5.49	0.05	0.7791
Breast muscle color					
Lightness (L*)	52.82	53.29	52.64	0.66	0.7739
Redness (a*)	10.64^b^	10.68^b^	11.05^a^	0.11	0.0270
Yellowness (b*)	8.56	8.47	8.64	0.06	0.2132
WHC, %	55.61	55.53	55.85	0.60	0.9253
Cooking loss	18.69	18.33	18.19	0.30	0.5032
Drip loss, %					
d1	4.14	4.37	4.19	0.16	0.5858
d3	7.06	7.21	7.12	0.07	0.3545
d5	9.55	9.68	9.66	0.07	0.4163
d7	12.22	12.16	12.13	0.08	0.7224
Relative organ weight, %					
Breast muscle	9.28^b^	9.60^a^	9.69^a^	0.09	0.0161
Liver	2.54	2.61	2.60	0.05	0.5959
Bursa of Fabricius	0.13	0.13	0.13	0.002	0.5293
Abdominal fat	3.57	3.54	3.48	0.07	0.5925
Spleen	0.17	0.16	0.17	0.005	0.9518
Gizzard	0.95	0.96	0.93	0.01	0.1773

^†^ Abbreviations: CON, basal diet; TRT1, basal diet + 0.1% DOL–60, and TRT2, basal diet + 0.1% DOL–97; WHC, water holding capacity. ^‡^ Standard error of the mean. ^a,b^ Means in the same row with different superscripts differ by the Tukey test (*p* < 0.05).

**Table 7:** The effect of dietary DOL supplementation on fecal microbial in broilers ^†,‡^.

Items, log_10_cfu/g	CON	TRT1	TRT2	SEM^3^	*p* Value
*Lactobacillus*	7.72	7.59	7.65	0.06	0.3910
*E. coli*	6.22^a^	6.18^ab^	6.13^b^	0.02	0.0391

^†^ Abbreviations: CON, basal diet; TRT1, basal diet + 0.1% DOL-60, and TRT2, basal diet + 0.1% DOL-97. ^‡^ Standard error of the mean. ^a,b^ Means in the same row with different superscripts differ by the Tukey test (*p* < 0.05).
